# Chemotypic Characterization and Biological Activity of *Rosmarinus officinalis*

**DOI:** 10.3390/foods6030020

**Published:** 2017-03-05

**Authors:** Prabodh Satyal, Tyler H. Jones, Elizabeth M. Lopez, Robert L. McFeeters, Nasser A. Awadh Ali, Iman Mansi, Ali G. Al-kaf, William N. Setzer

**Affiliations:** 1 Alchemy Aromatics LLC, 621 Park East Blvd., New Albany, IN 47150, USA; prabodhsatyal@gmail.com; 2 Department of Chemistry, University of Alabama in Huntsville, Huntsville, AL 35899, USA; tyler85@gmail.com (T.H.J.); eml0024@tigermail.auburn.edu (E.M.L.); robert.mcfeeters@uah.edu (R.L.M.); 3 Pharmacognosy Department, Faculty of Pharmacy, Sana’a University, P.O. Box 18084, Sana’a, Yemen; alinasser9678@yahoo.com or naali@bu.edu.sa (N.A.A.A.); alialkaf21@gmail.com (A.G.A); 4 Pharmacognosy Department, Faculty of Clinical Pharmacy, Albaha University, P.O. Box 1988, Al Baha, Saudi Arabia; 5 Department of Clinical pharmacy and Pharmacy Practice, Faculty of Pharmaceutical Sciences, The Hashemite University, P.O. Box 330127, Zarqa 13133, Jordan; man_mansi@hu.edu.jo

**Keywords:** essential oil, chemical composition, chiral gas chromatography, enantiomeric distribution, mass spectrometry, hierarchical cluster analysis, antifungal, cytotoxic, enzyme inhibition

## Abstract

Rosemary (*Rosmarinus officinalis* L.) is a popular herb in cooking, traditional healing, and aromatherapy. The essential oils of *R. officinalis* were obtained from plants growing in Victoria (Australia), Alabama (USA), Western Cape (South Africa), Kenya, Nepal, and Yemen. Chemical compositions of the rosemary oils were analyzed by gas chromatography-mass spectrometry as well as chiral gas chromatography. The oils were dominated by (+)-α-pinene (13.5%–37.7%), 1,8-cineole (16.1%–29.3%), (+)-verbenone (0.8%–16.9%), (−)-borneol (2.1%–6.9%), (−)-camphor (0.7%–7.0%), and racemic limonene (1.6%–4.4%). Hierarchical cluster analysis, based on the compositions of these essential oils in addition to 72 compositions reported in the literature, revealed at least five different chemotypes of rosemary oil. Antifungal, cytotoxicity, xanthine oxidase inhibitory, and tyrosinase inhibitory activity screenings were carried out, but showed only marginal activities.

## 1. Introduction

*Rosmarinus officinalis* L., “rosemary” (Lamiaceae) is an evergreen shrub with aromatic needle-like leaves. It is native to the Mediterranean and Asia, but is now cultivated in temperate locations around the world as a decorative garden plant and culinary herb. Rosemary leaves have been used to flavor foods such as lamb, pork, chicken, fish, and stuffings, and to prepare herbal oils, butters, and vinegars.

Rosemary has long been used in traditional medicine for a variety of conditions [1]. In the Mediterranean region, an infusion of the aerial parts is taken internally to treat colds and cough [2,3] as an antispasmodic, antihypertensive, and antiepileptic [4,5,6,7,8,9,10], to treat diabetes [6,9], and intestinal parasites [11]. A maceration of *R. officinalis* in alcohol or olive oil is used externally to treat contusions, rheumatism, and muscular and joint pains [3,12,13]. In Mexico, native peoples inhale the smoke from the burning plant to treat cough [14] or drink an infusion to treat vomiting, stomachache, or intestinal parasites [15]. Rosemary extracts contain a number of phytochemicals, including carnosic acid, carnosol, 12-*O*-methylcarnosic acid, rosmarinic acid, and genkwanin [16]. Rosemary essential oils contain 1,8-cineole [17], α-pinene [18], verbenone [19], camphor, and borneol [20], but the compositions can vary widely. There are several varieties of *R. officinalis*. The Missouri Botanical Garden currently lists 16 sub-taxa [21] and numerous cultivars have been developed as well.

Rosemary essential oil is used in aromatherapy as a nerve stimulant for purposes including memory loss and lethargy [22]. Rosemary oil has shown psychostimulant activity. In a mouse model, a dose of 100 μg/kg caused a significant increase in locomotor activity [23]. In humans, massaged [24] or inhaled [25] rosemary oil has caused significant increased blood pressure, heart rate, and respiratory rate; oral administration of rosemary oil significantly increased blood pressure in hypotensive patients [26]. Inhaled rosemary oil has been shown to increase memory and concentration abilities [27]. In addition to psychostimulatory effects, rosemary essential oil has shown anticholinesterase [28], acaricidal [29,30], antibacterial [31,32], antifungal [33,34], and antinociceptive [35] activities.

The biological activities of *R. officinalis* essential oils doubtless depend on the chemical compositions, and at least 13 different rosemary oil chemotypes have been previously identified, based on the relative percentages of α-pinene, 1,8-cineole, camphor, borneol, verbenone, and bornyl acetate [31,36,37,38,39,40,41]. In this work, we have characterized the essential oils of *R. officinalis* collected from Alabama (USA), Western Cape (South Africa), Victoria (Australia), Kenya, Nepal, and Yemen, and screened these essential oils for antifungal activity. In addition, a hierarchical cluster analysis has been carried out based on the compositions of an additional 72 rosemary essential oils reported in the literature.

## 2. Materials and Methods

### 2.1. Plant Material

Leaves of *R. officinalis* from flowering plants in Huntsville, Alabama (34°38′27.2″ N, 86°33′44.4″ W, elevation 183 m above sea level (asl)) were identified by W.N. Setzer, and collected on 4 August 2016. The fresh plant material (55.44 g) was hydrodistilled for 4 h using a Likens-Nickerson apparatus with continuous extraction with dichloromethane to give 950 mg (1.7% yield) colorless essential oil, which was stored at −20 °C until analysis.

The leaves of *R. officinalis* were collected during the flowering stage in May 2013, from Dhamar province, Yemen. The plant was identified by Dr. Hassan M. Ibrahim of the Botany Department, Faculty of Sciences, Sana’a University. A voucher specimen of the plant material (YMP-lam-31) has been deposited at the Pharmacognosy Department, Sana’a University, Yemen. The dried leaves were hydrodistilled for 3 h in a Clevenger type apparatus according to the European Pharmacopoeia [42]. The obtained oil was subsequently dried over anhydrous Na_2_SO_4_ and kept at 4 °C until analysis. After filtration, the yield of the oil was 1.1% *w*/*w*.

*R. officinalis*, in the flowering stage, from Jumla, Nepal (29°16′28.99″ N, 82°11′1.79″ E, elevation 2500 m asl), was identified by Prasun Satyal, and collected on 2 July 2016. The fresh plant material (100 g) was hydrodistilled for 4 h using a Clevenger-type apparatus and collected to give 500 mg (0.5% yield) colorless essential oil after drying with Na_2_SO_4_.

*R. officinalis* from Ribeeck Kasteel, Western Cape, South Africa (33°23′7.21″ S, 18°53′54.65″ E, elevation 300 m asl), was identified by Prabodh Satyal, and collected on 20 August 2016. The fresh plant material (500 g) was subjected to steam distillation for 4 h using a Clevenger-type apparatus and collected to give 4 g (0.8% yield) colorless essential oil after drying with Na_2_SO_4_.

Flowering *R. officinalis* from Lancefield, Victoria, Australia (37°16′ S, 144°43′ E, elevation 495 m asl), was identified by Chris Burder, and collected on 20 December 2015. The fresh plant material (1 kg) was subjected to steam distillation for 3 h using a Clevenger-type apparatus and collected to give 9 g (0.9% yield) colorless essential oil after drying with Na_2_SO_4_.

*R. officinalis* from Thika, Kenya (1°1′ S, 37°5′ E, elevation 1500 m asl), was identified by Aaron Sorensen, and collected on 8 July 2016. The fresh plant material (1 kg) was subjected to steam distillation for 3.5 h using a Clevenger-type apparatus and collected to give 10 g (1.0% yield) pale yellow essential oil after drying with Na_2_SO_4_.

### 2.2. Gas Chromatography-Mass Spectrometry (GC-MS)

The essential oils of *R. officinalis* were analyzed by GC-MS using a Shimadzu GCMS-QP2010 Ultra operated in the electron impact (EI) mode (electron energy = 70 eV), scan range = 40–400 atomic mass units, scan rate = 3.0 scans/s, and GC-MS solution software. The GC column was a ZB-5 fused silica capillary column with a (5% phenyl)-polymethylsiloxane stationary phase and a film thickness of 0.25 μm. The carrier gas was helium with a column head pressure of 552 kPa and flow rate of 1.37 mL/min. Injector temperature was 250 °C and the ion source temperature was 200 °C. The GC oven temperature program was programmed for 50 °C initial temperature, temperature increased at a rate of 2 °C/min to 260 °C. A 5% *w*/*v* solution of the sample in CH_2_Cl_2_ was prepared and 0.1 μL was injected with a splitting mode (30:1). Identification of the oil components was based on their retention indices determined by reference to a homologous series of *n*-alkanes, and by comparison of their mass spectral fragmentation patterns with those reported in the literature [43], and stored in our in-house MS library.

### 2.3. Chiral Gas Chromatography-Mass Spectrometry

Chiral analysis of the essential oils was performed on a Shimadzu GCMS-QP2010S operated in the EI mode (electron energy = 70 eV), scan range = 40–400 amu, scan rate = 3.0 scans/s. GC was equipped with a Restek B-Dex 325 capillary column (30 mL × 0.25 mm ID × 0.25 μm film). Oven temperature was started at 50 °C, and then gradually raised to 120 °C at 1.5 °C/min. The oven was then raised to 200 °C at 2 °C/min and held for 5 min. Helium was the carrier gas and the flow rate was maintained at 1.8 mL/min. Samples were diluted 3% *w*/*v* with CH_2_Cl_2_ and then a 0.1 μL sample was injected in a split mode with a split ratio of 1:45.

### 2.4. Hierarchical Cluster Analysis

A total of 72 *R. officinalis* essential oil compositions from the published literature [17,18,19,20,28,29,30,31,32,33,34,35,36,37,39,41,44,45,46,47,48,49,50,51,52,53,54,55,56,57,58,59,60,61,62,63,64,65,66,67,68,69,70,71,72,73,74,75,76,77,78,79,80,81] in addition to the six samples from this study were treated as operational taxonomic units (OTUs). The percentage composition of 23 major essential oil components (1,8-cineole, α-pinene, camphor, verbenone, borneol, camphene, myrcene, bornyl acetate, β-pinene, limonene, α-terpineol, linalool, β-caryophyllene, *p*-cymene, terpinen-4-ol, γ-terpinene, geraniol, α-phellandrene, terpinolene, α-terpinene, α-humulene, sabinene, and caryophyllene oxide) was used to determine the chemical relationship between the various *R. officinalis* essential oil samples by agglomerative hierarchical cluster (AHC) analysis using the XLSTAT software, version 2015.4.01 (Addinsoft™, New York, NY, USA). Pearson correlation was selected as a measure of similarity, and the unweighted pair-group method with arithmetic average (UPGMA) was used for cluster definition.

### 2.5. Antifungal Screening

Antifungal activity was carried out as previously described [82]. Briefly, minimum inhibitory concentrations were determined using microdilution methods for *Candida albicans* (American Type Culture Collection, ATCC #18804), *Cryptococcus neoformans* (serotype D or var. *neoformans*) (ATCC #24067), and *Aspergillus niger* (ATCC #16888). Single colonies from potato dextrose agar plates were grown in potato dextrose broth for three days. Cells were diluted to 2 × 10^3^ cells/mL using MOPS (3-(*N*-morpholino)propanesulfonic acid) buffered RPMI (Roswell Park Memorial Institute) medium and aliquoted into sterile 12 × 75 mm tubes (900 μL). Essential oil (100 μL) was added to each tube followed by incubation at 37 °C for three days in a shaking incubator (175 rpm). Minimum inhibitory concentrations (MICs) were calculated from microdilution in 96-well plates, performed in triplicate. Serial dilution was performed by adding 50 μL of RPMI to each well, then an equal volume of essential oil to be tested to the first row. After mixing, 50 μL was removed and added to the next row. The procedure was repeated for each row, discarding the final 50 μL removed. To the mixture in each well, 50 μL of cells were added. The plates were incubated for two days at 37 °C before growth was quantitated from turbidity.

### 2.6. Xanthine Oxidase Inhibition Assay

Using xanthine as substrate, the xanthine oxidase (XO) activity of *R. officinalis* oil from Yemen was assayed spectrophotometrically according to Apaya and Hernandez [83]. A mixture containing 1 mL of 100 μg/mL of *R. officinalis* oil or allopurinol, 1.9 mL of 50 mM potassium phosphate buffer, and 1 mL of xanthine substrate (0.6 mM) was preincubated for 10 min at 25 °C, and the reaction was started by the addition of 0.1 mL of XO enzyme (0.1 U/mL in phosphate buffer). The reaction was incubated at 25 °C for 30 min, and the absorbance was measured against phosphate buffer as blank at 295 nm using quartz cuvettes. Allopurinol was used as standard enzyme inhibitors. The percent xanthine oxidase inhibition was calculated according to the following formula:

% inhibition = 100 − (A_1_ − B) × 100/(A_o_ − B) (1)

where A_1_ is the activity of the enzyme in presence of the oil, B is the absorbance in absence of the enzyme, and A_o_ is the absorbance in absence of the oil or inhibitor.

### 2.7. Tyrosinase Inhibition Assay

In vitro mushroom tyrosinase inhibitory activity of *R. officinalis* oil from Yemen was determined by a spectrophotometric approach using l-tyrosine as the substrate [84]. In a total volume of 200 µL, the enzyme activity was measured in buffer containing 50 mM phosphate buffer, pH 6.5, 50 U/mL mushroom tyrosinase, and 50 µg/mL l-tyrosine. The reaction (conversion of l-tyrosine to DOPAchrome) was conducted at 37 °C for 30 min, and absorbance was then measured at the wavelength of 490 nm using a microplate reader. Blanks were run for the same concentration of essential oil in the absence of the enzyme. The inhibition assays were carried out in the presence of 100 µg/mL of the essential oil. Kojic acid and arbutin were used as positive control inhibitors in these assays. The absorbance of the same mixture without the essential oil was used as the negative control. The percent inhibition of tyrosinase activity was calculated as follows:

% inhibition = ((A − B)/A) × 100(2)

where A is the absorbance difference at 490 nm of the negative control (enzyme activity without oil)—the absorbance of the blank (no enzyme, no oil); B is the absorbance of test sample (with enzyme)—the absorbance of test sample blank (no enzyme).

### 2.8. Cytotoxicity Screening

Human colorectal cancer cell lines (SW480 and HCT116) were generously provided by Dr. Rick F. Thorne (University of Newcastle, Australia) and were cultured in Dulbecco’s Modified Eagle Medium (DMEM) containing 10% fetal calf serum (Bio Whittaker, Verviers, Belgium). The rosemary oil from Yemen was tested for acute cytotoxic effect; the essential oil was dissolved in dimethylsulfoxide (DMSO) and different dilutions in culture buffer were prepared. The acute cytotoxic effect of the essential oil on colorectal cancer cells was determined using the MTT (3-(4,5-dimethylthiazol-2-yl)-2,5-diphenyltetrazolium bromide) assay [85]. Briefly, cells were seeded at 5000 cells per well onto flat-bottomed 96-well culture plates and allowed to grow for 24 h before the desired treatment. Cells were then incubated with 200 µL of essential oil concentrations (0–200 μg/mL) for 72 h. Cells were then labeled with MTT from the Vybrant MTT Cell Proliferation Assay Kit (Molecular Probes, Eugene, OR, USA) according to the manufacturer’s instruction and resulting formazan was solubilized with DMSO. Absorbance was read in a microplate reader at 540 nm.

## 3. Results and Discussion

### 3.1. Essential Oil Compositions

The chemical compositions of six *Rosmarinus officinalis* essential oils are compiled in Table 1. α-Pinene and 1,8-cineole dominated the essential oils of all six samples (13.5%–38.1% and 16.3%–29.4%, respectively). Verbenone was also relatively abundant in the samples from Alabama (USA), Kenya, and Yemen. The sample from Yemen, with its relatively high verbenone content (18.6%) and relatively low α-pinene content (13.5%), along with 1,8-cineole (20.6%) and camphor (7.0%), firmly places it in the *verbenoniferum* chemotype, type IIIA, according to Napoli et al. [39]. The other five samples in this study have higher concentrations of α-pinene than 1,8-cineole or verbenone, and can be characterized, based on Napoli et al., as *cineoliferum* type VIA [39].

The enantiomeric distributions of monoterpenoids of the rosemary oils, determined by chiral GC-MS, are summarized in Table 2. (+)-α-Pinene was the predominant enantiomer (97%–99%) in the five samples analyzed in this study. β-Pinene, on the other hand, showed 29%–33% (+)-enantiomer, in general agreement with that reported by Presti and co-workers, 36%–40% (+)-β-pinene [86]. The enantiomeric distributions of limonene (40%–54% (+)-limonene), linalool (95%–97% (−)-linalool), terpinen-4-ol (69%–70% (+)-terpinen-4-ol), and α-terpineol (66%–75% (+)-α-terpineol), are also in qualitative agreement with those reported by Presti et al., 48%–53% (+)-limonene, 99% (−)-linalool, 73%–76% (+)-terpinen-4-ol, and 66%–67% (+)-α-terpineol [86]. Presti and co-workers found (+)-sabinene to be dominant in rosemary oil (96%–98%) [86], but sabinene concentrations were too low to determine the enantiomeric distribution in the samples in this study. König and co-workers found the enantiomeric distribution of borneol in rosemary oils to show significant differences; the (−)-enantiomer generally dominated, but was much larger in rosemary oil originating in Spain [87]. In this current study, (−)-borneol also dominated (95%–98%). Furthermore, bornyl acetate in this study was enantiomerically pure with 100% (−)-bornyl acetate. Verbenone was also enantiomerically pure, 100% (+)-verbenone, in all rosemary oils examined. Ravid and co-workers also found high enantiomeric purity (96%–100% (+)-verbenone) in their *R. officinalis* essential oil samples [88]. Thus, we can conclude that the (+)/(−) ratios of each of the monoterpenoids remains relatively constant in rosemary oils, regardless of geographical location of the oil source.

### 3.2. Chemotypes of Rosemary

**Figure 1:**
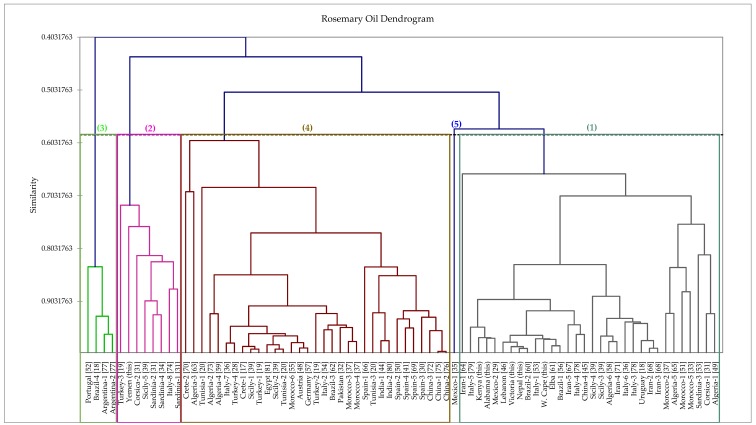
Dendrogram obtained from the agglomerative hierarchical cluster analysis of 78 *Rosmarinus officinalis* essential oil compositions.

In order to provide additional insight into the chemotypes of rosemary essential oils, we have carried out a hierarchical cluster analysis based on the chemical compositions of the six oils in this study along with 72 additional rosemary oil chemical compositions from the literature. The dendrogram of the analysis is shown in Figure 1. Based on this analysis, there are five different chemotypes: (1) α-pinene/1,8-cineole, (2) verbenone/α-pinene/camphor/1,8-cineole, (3) myrcene/1,8-cineole/camphor, (4) 1,8-cineole/camphor/α-pinene, and (5) α-pinene/β-pinene/camphene. Chemotype 1, dominated by α-pinene [36,37,89,90,91,92], is a cluster made up of 32 samples, including those from Victoria (Australia), Nepal, Kenya, Western Cape (South Africa), and Alabama (USA). Chemotype 2, dominated by verbenone (i.e., the *verbenoniferum* chemotype) [39,74], has eight samples, including the sample from Yemen. Chemotype 3, dominated by myrcene [89], has four samples in this analysis. Chemotype 4 is dominated by 1,8-cineole [36,38,93,94,95] and is comprised of 33 samples, and can be considered comparable to the *cineoliferum* chemotype described by Napoli and co-workers [39]. The fifth chemotype, represented by only one sample from Mexico [35], has nearly equal quantities of α-pinene, β-pinene, and camphene; the relatively high β-pinene concentration (12%) separates this sample from chemotype 1.

### 3.3. Antifungal Activity

Antifungal activity was tested against three common opportunistic fungal pathogens. Yeast-like *C. albicans* and mold-like *A. niger* are Ascomycota while *C. neoformans* is a Basidiomycota. Overall, no inhibitory activity at or below 2500 ppm for the *R. officinalis* essential oil chemotypes was overserved against either ascomycete. Inhibitory activity was observed against *C. neoformans* for the majority of *R. officinalis* chemotypes tested. Though not exceedingly promising, *R. officinalis* from Australia did demonstrate an MIC of 625 ppm. Previous investigations of antifungal activity rosemary oil against *A. niger* [76,96,97] and *C. albicans* [19,98,99] have also found rosemary oil to be inactive, while against *C. neoformans* it had been found to be moderately active (MIC = 156 μg/mL) [100]. Thus, rosemary oil cannot be considered a worthwhile antifungal agent.

### 3.4. Other Biological Assays

The rosemary oil from Yemen was screened for xanthine oxidase inhibitory, tyrosinase inhibitory, and cytotoxic (SW480 and HCT116 human colorectal carcinoma cells) activity, but showed no activity in any of these assays. Yemeni rosemary oil showed only 8.6% ± 4.7% inhibition of xanthine oxidase. Against tyrosinase, Yemeni rosemary oil showed only 3.3% ± 1.5% inhibition at 100 μg/mL. In contrast, rosemary oil from cultivated plants from Mauritius did show tyrosinase inhibitory activity (median inhibitory concentration, IC_50_ = 97 μg/mL) [101]. Rosemary oil was found to be relatively non-toxic to SK-OV-3, HO-8910, Bel-7402 (IC_50_ ≥250 μg/mL) [102], MCF-7, LNCaP, and NIH-3T3 cells (IC_50_ >180 μg/mL) [32], but active against A549 cells (IC_50_ = 80 μg/mL) [103]. In summary, the enzyme inhibitory potential of rosemary oil is fairly low. In high concentrations, however, rosemary oil may be harmful to some mammalian tissues.

## 4. Conclusions

Rosemary (*Rosmarinus officinalis*) essential oil has been extensively studied for its chemical composition and its biological activities. α-Pinene and 1,8-cineole generally dominate the essential oil compositions, but camphor, verbenone, camphene, and myrcene may also appear in high concentrations. The enantiomeric distributions of monoterpenoids in rosemary oils seem to remain relatively constant regardless of environmental factors. Based on the relative concentrations of the major components in rosemary oils, we can define at least five separate chemotypes. The different chemotypes are likely to present different biological activities, but rosemary oil, in general, is relatively inactive in terms of antifungal, enzyme inhibitory, or antitumor potential.

## Figures and Tables

**Table 1:** Chemical compositions of essential oils of *Rosmarinus officinalis* from six different geographical locations.

RI	Compound	Percent Composition					
		Alabama	Western Cape	Kenya	Victoria	Nepal	Yemen
800	*n*-Octane	--- ^a^	---	---	tr ^b^	---	---
801	Hexanal	tr	---	---	---	---	---
849	(2*E*)-Hexenal	tr	---	---	---	---	---
850	(3*Z*)-Hexenol	tr	---	0.1	---	---	---
920	Artemisia triene	---	---	---	tr	---	---
922	Tricyclene	0.1	0.4	0.1	0.2	0.2	0.1
925	α-Thujene	0.1	0.1	0.2	0.1	0.2	0.2
934	α-Pinene	25.4	33.6	31.7	37.9	38.1	13.5
941	Thujadiene	---	---	---	---	---	tr
947	α-Fenchene	tr	0.2	tr	tr	0.1	---
950	Camphene	2.5	7.6	2.6	4.6	4.6	1.5
953	Thuja-2,4(10)-diene	0.5	1.2	0.4	0.8	0.8	0.3
972	Sabinene	tr	---	0.1	tr	0.1	tr
978	β-Pinene	1.4	2.3	2.1	3.0	2.8	1.0
890	1-Octen-3-ol	---	0.6	---	---	---	0.1
984	6-Methyl-5-Hepten-2-one	---	---	tr	tr	tr	---
984	3-Octanone	---	0.2	---	---	---	tr
989	Myrcene	1.3	1.6	1.2	1.5	1.4	0.7
989	Dehydro-1,8-cineole	---	---	0.1	---	---	---
1004	*p*-Mentha-1(7),8-diene	---	---	---	tr	tr	---
1005	α-Phellandrene	0.1	0.1	0.2	0.2	0.2	---
1009	δ-3-Carene	---	---	---	---	0.1	0.2
1017	α-Terpinene	0.3	0.3	0.4	0.5	0.6	tr
1019	*p*-Cymene	0.9	1.6	0.4	1.6	1.5	1.1
1029	Limonene	2.7	4.4	2.3	3.5	3.5	1.6
1031	1,8-cineole	18.8	16.3	20.9	29.4	23.0	20.6
1035	(*Z*)-β-Ocimene	---	0.6	tr	tr	tr	---
1044	(*E*)-β-Ocimene	---	0.2	---	---	---	---
1048	Thujol	---	---	---	---	---	0.1
1058	γ-Terpinene	1.0	0.4	1.0	0.8	1.2	0.1
1069	*cis*-Sabinene hydrate	0.1	tr	0.3	tr	---	0.4
1070	*cis*-4-Thujanol	---	---	---	---	tr	---
1085	Terpinolene	0.7	0.3	0.8	0.5	0.7	0.2
1086	*trans*-Linalool oxide (furanoid)	---	---	---	---	---	0.1
1090	*p*-Cymenene	tr	0.1	tr	0.1	0.1	---
1091	Rosefuran	---	---	---	tr	---	---
1098	Linalool	2.7	3.1	2.8	2.3	2.2	3.8
1098	*cis*-Decahydronaphthalene + Perillene	---	---	---	---	---	0.1
1100	*trans*-Sabinene hydrate	---	---	0.2	---	---	---
1103	Hotrienol	---	---	---	tr	---	---
1105	(2*E*)-Hexenyl propanoate	---	---	---	tr	---	---
1106	Isochrysanthenone	tr	0.1	tr	tr	tr	---
1117	*endo*-Fenchol	tr	0.1	tr	tr	tr	tr
1117	2,4-Dimethyl-2,4 heptadienal	---	---	---	---	---	0.1
1122	Chrysanthenone	0.2	0.5	0.5	0.5	0.3	0.5
1123	*cis-p*-Menth-2-en-1-ol	tr	---	tr	tr	---	---
1126	α-Campholenal	0.1	0.1	0.1	0.1	0.1	0.1
1139	*trans*-Pinocarveol	0.1	0.5	---	0.1	0.1	0.1
1141	*cis*-Verbenol	0.2	0.1	0.3	0.1	tr	0.2
1144	*trans*-Verbenol	0.4	0.2	0.5	0.1	0.1	---
1146	Camphor	2.4	0.7	2.1	1.7	1.7	7.0
1154	*trans*-β-Terpineol	tr	0.1	tr	---	tr	tr
1155	Camphene hydrate	---	---	---	tr	---	---
1161	*trans*-Pinocamphone	0.2	0.4	0.1	0.1	0.1	0.2
1162	Pinocarvone	0.3	0.1	0.3	0.2	0.1	0.2
1165	Isofenchol	0.2	tr	0.2	tr	0.1	---
1169	δ-Terpineol	0.3	---	0.3	0.1	0.2	---
1171	Borneol	4.0	7.0	2.8	2.1	2.5	5.5
1175	*cis*-Pinocamphone	0.7	1.1	0.6	0.4	0.4	0.9
1177	*p*-1,8-Menthadien-4-ol	tr	tr	tr	tr	---	tr
1179	Terpinen-4-ol	1.1	0.7	1.0	0.5	2.2	1.0
1185	*p*-Cymen-8-ol	0.1	tr	0.1	tr	tr	0.3
1194	α-Terpineol	2.9	1.0	2.6	0.9	1.5	3.2
1197	Methyl chavicol	tr	---	tr	tr	tr	---
1206	Verbenone	17.1	0.8	11.9	2.5	2.7	18.6
1213	3-Oxo-1,8-cineole	0.1	---	0.1	tr	---	tr
1218	(4-Methylpentyl)-cyclohexane	0.4	---	0.2	---	tr	0.2
1218	*trans*-Carveol	---	---	---	tr	---	---
1225	Citronellol	0.3	---	0.3	tr	0.2	0.3
1228	Bornyl formate.	---	---	---	tr	---	---
1238	Neral	0.1	---	0.2	tr	0.1	0.1
1239	*cis*-Shisool	0.3	0.3	0.1	tr	0.1	0.3
1242	Carvone	0.1	---	tr	tr	tr	tr
1242	Hexyl isovalerate	---	---	---	---	tr	---
1246	*trans*-Shisool	0.4	0.3	0.2	tr	0.1	0.5
1247	Carvotanacetone	---	---	---	---	---	3.0
1250	Geraniol	4.8	---	4.6	0.7	1.6	3.8
1252	*cis*-Myrtanol	0.1	---	tr	---	---	0.1
1257	Methyl citronellate	---	---	---	---	tr	---
1267	Geranial	0.1	---	0.2	0.1	0.1	---
1268	Isopiperitenone	0.1	0.1	0.2	---	0.1	0.3
1280	*cis*-Verbenyl acetate	---	tr	---	tr	---	0.1
1284	Bornyl acetate	1.7	2.0	1.0	0.9	1.0	1.8
1290	Thymol	---	---	---	---	---	0.5
1296	Carvacrol	tr	---	---	---	---	---
1297	Perilla alcohol	tr	---	---	---	---	---
1297	Geranyl formate + Carvacrol	---	---	---	---	---	0.1
1321	Myrtenyl acetate	tr	---	tr	---	---	---
1321	Methyl geranate	---	---	---	---	tr	---
1332	δ-Elemene	0.1	---	---	tr	---	---
1332	*cis*-Piperitol acetate	---	---	tr	---	tr	tr
1337	Piperitenone.	tr	---	0.1	---	tr	0.1
1348	Citronellyl acetate	tr	---	---	---	---	---
1349	Eugenol	0.1	---	---	---	---	---
1361	Neoisodihydrocarvyl acetate	---	---	---	---	---	tr
1367	Linalyl isobutanoate	0.1	---	---	tr	---	
1368	α-Ylangene	---	0.2	---	---	---	---
1174	α-Copaene	---	0.8	---	---	---	0.1
1378	Geranyl acetate	0.2		0.2	tr	0.1	0.3
1392	2-Ethylidene-6-methyl-3,5-heptadienal	---	0.1	0.2	0.1	---	---
1400	Methyleugenol	0.4	0.2	0.2	tr	0.1	0.2
1419	β-Caryophyllene	0.6	2.5	0.7	1.3	1.4	1.2
1427	β-Gurjunene	---	---	---	---	---	0.1
1431	α-Maaliene	---	---	---	---	---	tr
1438	Aromadendrene	---	0.2	---	---	---	---
1448	Geranyl acetone	tr	0.1	tr	---	---	0.1
1454	α-Humulene	0.1	0.5	0.1	0.1	0.2	0.2
1472	*cis*-Cadina-1(6),4-diene	---	0.1	---	---	---	---
1475	*trans*-Cadina-1(6),4-diene	---	0.7	---	---	tr	0.1
1481	*ar*-Curcumene	---	tr	---	---	---	---
1488	β-Selinene	---	0.1	---	---	---	---
1492	γ-Amorphene	---	0.1	---	---	---	---
1496	α-Selinene	---	0.2	---	---	---	---
1499	α-Muurolene	---	0.3	---	---	0.1	---
1503	Valencene.	---	0.1	---	---	---	---
1509	β-Bisabolene	---	0.1	---	---	---	---
1513	δ-Amorphene	---	0.6	---	---	0.1	tr
1516	δ-Cadinene	---	1.0	---	---	0.3	0.1
1522	*trans*-Calamenene	---	0.2	---	---	---	---
1533	*trans*-Cadine-1,4-diene	---	0.1	---	---	---	---
1537	α-Cadinene	---	0.1	---	---	---	---
1541	α-Calacorene	---	0.1	---	---	---	---
1548	Elemol	---	---	---	---	0.5	---
1566	Maaliol	---	---	---	---	---	1.6
1573	Spathulenol	---	---	---	---	---	0.1
1582	Caryophyllene oxide	0.2	0.3	0.2	tr	0.1	0.5
1582	Gleenol	---	---	---	---	---	tr
1608	Humulene epoxide II	tr	0.1	---	---	---	tr
1631	γ-Eudesmol	---	---	---	---	0.1	---
1635	Caryophylla-4(12),8(13)-dien-5-ol	tr	---	---	---	---	---
1654	14-Hydroxy-9-*epi*-(*Z*)-Caryophyllene	0.1	0.1	---	---	---	---
1655	α-Eudesmol	---	---	---	---	0.2	---
1683	α-Bisabolol	---	---	---	---	---	tr
1764	Benzyl benzoate	tr	---	---	---	---	---
1777	8α-Acetoxyelemol	---	---	---	---	tr	---
	Total Compounds Identified	53	66	52	36	52	59
	Percent Composition Identified	99.1	99.7	99.5	99.9	99.7	98.8

^a^ --- = not detected. ^b^ tr = trace (<0.05%).

**Table 2:** Enantiomeric distribution of monoterpenoids in *Rosmarinus officinalis* essential oils from six different geographical locations.

Compound	Enantiomeric Distribution, (+)/(−)				
	Alabama	Western Cape	Kenya	Victoria	Nepal
α-Pinene	99/1	99/1	97/3	99/1	98/2
Camphene	75/25	78/22	75/25	75/25	75/25
β-Pinene	33/67	29/71	31/69	30/70	30/70
Limonene	48/52	40/60	53/47	54/46	54/46
Linalool	5/95	3/97	5/95	3/97	5/95
Camphor	15/85	16/84	15/85	15/85	15/85
Borneol	2/98	2/98	5/95	5/95	4/96
Terpinen-4-ol	69/31	70/30	70/30	70/30	70/30
α-Terpineol	74/26	75/25	71/29	68/32	66/34
Verbenone	100/0	100/0	100/0	100/0	100/0
Bornyl acetate	0/100	0/100	0/100	0/100	0/100
